# The Efficacy of Honey for the Treatment of Perineal Wounds Following Vaginal Birth: A Narrative Review

**DOI:** 10.3390/ph18020182

**Published:** 2025-01-29

**Authors:** Isa S. Schaap, Céline M. J. G. Lardenoije, Senna J. J. M. van Riel, Niels A. J. Cremers

**Affiliations:** 1 Department of Gynecology and Obstetrics, Maastricht University Medical Centre+, P. Debyelaan 25, 6229 HX Maastricht, The Netherlands; isaschaap.research@gmail.com (I.S.S.); celine.lardenoije@mumc.nl (C.M.J.G.L.); sjjmvanriel@gmail.com (S.J.J.M.v.R.); 2 VieCuri Medical Centre, Tegelseweg 210, 5912 BL Venlo, The Netherlands; 3 GROW Research Institute for Oncology and Reproduction, Universiteitssingel 40, 6229 ER Maastricht, The Netherlands; 4 Triticum Exploitatie BV, Sleperweg 44, 6222 NK Maastricht, The Netherlands

**Keywords:** birth, perineal trauma, episiotomy, perineal tears, honey, medical grade honey, wound healing, pain

## Abstract

**Background/Objectives**: During vaginal delivery, the perineum can be damaged either by episiotomy or by a spontaneous perineal tear, leading to several complications. The wound healing process should proceed as quickly and properly as possible without an infection. Medical grade honey (MGH) may be a potent treatment option due to its antimicrobial and pro-healing activities. This literature study investigated the role of honey in the treatment of vaginal wounds after delivery. **Methods**: Studies published before 17 July 2024 in the PubMed, Web of Science, Embase, Scopus, EBSCO host/CINAHL, Cochrane Library, and Google Scholar databases about honey, episiotomy wounds, and perineal tears, as well as those investigating wound healing and/or pain, were assessed. **Results**: Ten studies were included (six RCTs, of which three were double-blind, one was quasi-experimental with a posttest only, and three were observational studies without a control group), with 723 participants in total. Six of the seven controlled studies showed honey significantly improved various outcome measures, such as improved wound healing, and reduced need for pain medication. The three non-controlled studies also had a positive outcome, improving wound healing and decreasing pain intensity and prickling sensation. However, the overall quality of available evidence is limited. Different types of honey concentrations, origins, and additives were used in the included studies. Using a standardized MGH formulation may help to maintain consistent and potent effects. Therefore, additional research is needed to determine the efficacy of MGH in perineal trauma and to establish guidelines for clinical use. **Conclusions**: Honey potentially has a great effect on wound healing of perineal trauma; however, more research is necessary to substantiate the findings in the current literature.

## 1. Introduction

During vaginal delivery, the perineum can be damaged either by episiotomy or by a spontaneous perineal tear. At least 85% of women undergoing vaginal delivery suffer from perineal trauma of varying degrees [1,2]. In the UK, only 8.6% of the nulliparous women who give birth in the hospital have an intact perineum, and 28.5% of the multiparous women [3]. The episiotomy rate in vaginal deliveries varies worldwide, from 91% in Thailand to 4% in Denmark [4]. In the Netherlands, 16% of women who gave birth received an episiotomy in 2020 [5].

An episiotomy is a surgical procedure in which an incision is made in the perineum in the final stage of labor to dilate the vaginal orifice [6,7]. Various types of incisions are performed. In Europe, the mediolateral incision is the most common, and in the United States, the midline episiotomy is [6,7]. Indications for an episiotomy are fetal distress and shoulder dystocia [7,8]. Perineal tears can be divided into four grades: Grade 1 is a laceration limited to the vaginal mucosa or perineal skin; grade 2 involves the perineal muscles; grade 3 also includes the anal sphincter muscles; and grade 4 extends through the rectal mucosa [9,10]. In the literature, it remains unclear whether episiotomies could be used to prevent third- and fourth-degree tears, as the studies supporting this claim are observational and may be affected by confounding [9,11]. Routine use of episiotomies is not recommended by the World Health Organization [12] and the American College of Obstetrics and Gynecologists [13].

Perineal trauma is associated with both short- and long-term complications, such as pain and increased infection rates, which can lead to wound dehiscence [2,13,14,15,16,17]. Up to 80% of the dehiscence of episiotomy wounds are due to infection [18]. The incidence of infection after perineal trauma varies from 0.1% to 23.6% and wound dehiscence occurs between 0.21% to 24.6% [19]. Some studies show that severe perineal trauma is associated with anal incontinence, dyspareunia, and delays in resuming sexual activity [16,17,20,21,22]. Additionally, there might be an association between postpartum physical symptoms, such as pain and infection, and postnatal depression, anxiety, or posttraumatic stress symptoms [2]. Proper treatment is crucial to prevent an infection and accelerate recovery.

Honey may be a potent treatment to accelerate healing and has been used as therapy for wounds and local infections for thousands of years. It is an easily accessible, affordable option with good healing properties [23,24]. Honey is a thick, viscous substance composed of water, sugars, proteins, amino acids, enzymes (e.g., glucose oxidase), beeswax, minerals, pigments, and pollen [25]. Medical-grade honey (MGH) differs from regular honey because it is carefully assessed to ensure the quality of the honey, its safety, and efficacy. MGH is free of pollutants like pesticides, herbicides, heavy metals, and antibiotics [26].

MGH has antimicrobial and pro-healing properties. MGH promotes wound healing by multiple mechanisms, often related to its physicochemical composition. Its physical viscous state creates a moist wound environment, ensuring faster healing than dry or wet wounds. Autolytic debridement is stimulated by the low pH, the activation of proteases, and the lymphatic outflow generated by the osmotic activity. Granulation tissue formation, angiogenesis, and epithelialization are enhanced by the high sugar content serving as a nutrient source for the proliferation and migration of skin cells. Edema is reduced by honey’s anti-inflammatory properties and osmotic activities attracting lymph fluid from beneath the wound bed [23,24,27].

Honey’s antimicrobial mechanisms are also mediated by a multitude of mechanisms. Honey has a strong osmotic effect due to the high amount of sugar in honey, causing bacterial dehydration and inhibiting bacterial growth and cell division [23]. Glucose oxidase gradually converts glucose into gluconic acid and hydrogen peroxide. Gluconic acid causes the acidity of honey to reach a pH between 3.5 and 4.0, creating an environment inhospitable for bacterial growth [23]. Hydrogen peroxide aids in eliminating bacteria and sterilizes the wound site [24,25,27]. Phytomolecules in honey, such as flavonoids and polyphenols (e.g., methylglyoxal, B-defensin, chrysin), can have direct antimicrobial activity against planktonic bacteria and those existing in biofilms [23], or against viruses, fungi, and protozoa [27]. No resistance to honey has been reported to date because of the multitude of antimicrobial mechanisms [28]. Moreover, MGH possesses an immunomodulatory activity, having a dual role in controlling inflammation and infection [29]. In the inflammatory phase, honey can stimulate pro-inflammatory cytokines such as TNF-α, IL-1β, and IL-6, and activate B-, T-lymphocytes, and neutrophils that subsequently help to resolve infections. Contiguously, honey can downregulate NF-κβ and MAPK pathways and exert anti-inflammatory and antioxidant effects, stimulating wound healing and reducing scar formation [23,24,27,29]. MGH is considered safe in wound care, with minimal adverse effects. Honey allergy is very rare, with an estimated incidence of 1:100,000 people consuming honey [30]. Topical application of honey may be even less harmful than consuming it; however, it is not advised to use honey in people with a known allergy. Another side effect of honey may be experiencing a stinging or burning sensation upon application to injured tissue. This pain is thought to be caused by a strong osmotic effect, especially when high concentrations of honey are used. However, honey has also been reported to decrease pain levels during wound evolution [31]. Therefore, MGH may be a good therapy option for perineal wounds.

This study aims to conduct a literature search to investigate the clinical efficacy and the effects on experienced pain and wound healing of honey on episiotomy wounds or perineal tears.

## 2. Results

### 2.1. Study Selection

**Figure 1:**
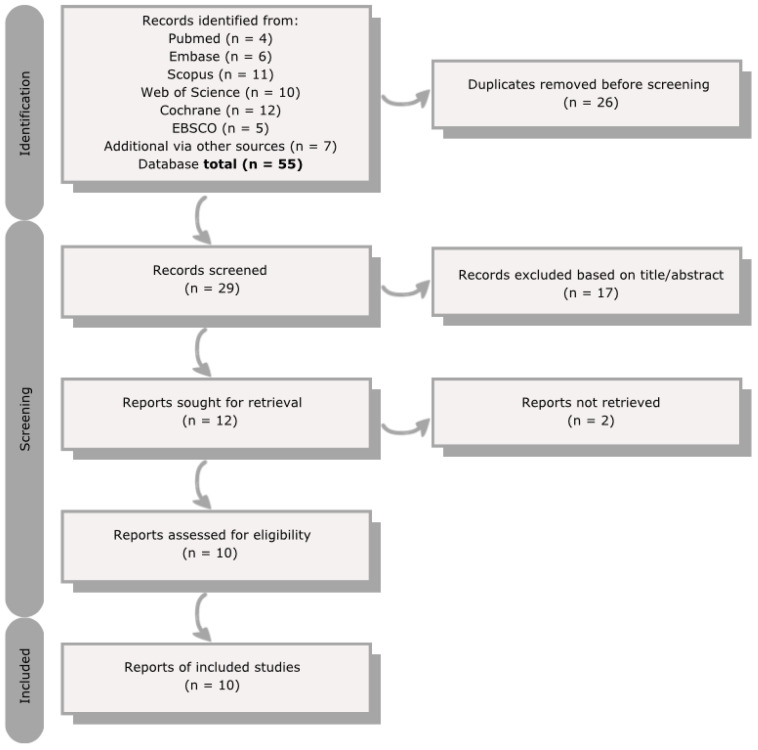
Flowchart study selection.

A systematic literature search was performed in databases PubMed, Embase, Cochrane, EBSCO host/CINAHL, Web of Science, and Scopus, followed by a free-text search on Google Scholar. More details on the search strategy, study selection, and data extraction can be found in the methods section. A total of 55 studies were identified from different databases, as demonstrated in the flowchart (Figure 1). The duplicates (*n* = 26) were removed before screening, and 29 articles remained. These remaining articles were screened, and 17 were excluded based on title and abstract when it was clear that the topic of the publication was unrelated. The full text of twelve reports was sought, of which two could not be retrieved (no full text available). In the end, 10 studies were included in this article.

### 2.2. Study Characteristics

The ten studies have a total of 723 participants who received an episiotomy or had a perineal tear. The number of participants per study ranged from 20 to 120. In three studies it remains unclear whether participants had perineal tears or episiotomies (*n* = 86). In the other studies, a total of 579 participants received an episiotomy and 50 participants had perineal tears. Due to the loss of follow-up in one of these studies [32], the perineal trauma status for eight participants remains unknown. Different outcome measures were examined; the majority of studies had episiotomy wound healing as the primary outcome measure, assessed through the REEDA-score (see Table 1). This scale can be used to evaluate the wound healing process by describing the following five parameters: redness, edema, ecchymosis, discharge, and approximation of perineal tissue [33,34]. A lower total score indicates better wound healing.

Five studies investigated the effect of honey on perineal pain intensity, assessed through the Visual Analogue Scale (VAS) for instance. Other outcome measures were pricking or burning sensation and tenderness (VAS), the number and dosage of pain relief drugs used, and the size of the wound (cm). Six study designs are randomized controlled trials (RCTs). Also, two comparative clinical study designs are used, a quasi-experimental posttest-only control group design and a one-group pretest–posttest design. Of all the studies, four took place in Iran, three in India, two in Indonesia, and one in Switzerland.

A clear overview of the included studies is provided in Table 2. Here, more detailed study characteristics such as the year of publication, country, study design, number of participants, inclusion and exclusion criteria, intervention and treatment details, control group, outcomes, and main conclusions are presented.

### 2.3. Data Synthesis

A meta-analysis could not be conducted due to the heterogeneity of studies. There are large variations in study design and intervention, e.g., honey formulation, dosing, application route, frequency of administration, and treatment duration, and differences in other study parameters, such as the use and composition of the control group, assessed outcome parameters, and evaluated time points (see Table 2). Due to the extensivity and diversity of the included studies, we created a new table to provide a quick overview of the results and enable a direct visual comparison of the studies (Table 3). The results can be divided into four key outcomes: wound healing, pain intensity, use of drugs to relieve pain, and pricking sensation. The significance level was included in the table depending on the study type having a control group.

The effect of honey on wound healing was investigated in nine of the ten included studies. Eight studies reported beneficial effects of honey, five of these studies were with a control group [35,36,37,38,39,40,42,43]. Torkashvand et al. [43] demonstrated a significantly lower mean REEDA score in the Olea ointment group (honey) compared to the control group at four intervals. The same applies to the honey group in the RCT by Lavaf et al. [38] on the 7th day postpartum. The RCT by Manjula et al. [39] showed a significantly faster healing pace with honey compared to betadine, and the RCT of Ghaderbasti et al. [36] proved that there was a significantly greater reduction in REEDA score in the honey group compared to the olive oil group. Mulyaningsih et al. [40] conducted a quasi-experimental, posttest-only control group study, in which the consumption of pineapple juice and honey had a significant effect on the acceleration of perineal wound healing. Nikpour et al. did not find a significant difference in wound healing when comparing the mean REEDA scores of the different treatment groups (honey, curcumin, and placebo) [41]. Jaiswal et al. and Pal et al. performed comparative clinical studies in which the outcome after treatment was compared to baseline levels [37,42]. Jaiswal found significant improvements in redness and edema, while Pal found significant improvements in redness, edema, and ecchymosis, both supporting a positive effect in wound healing. The one-group pretest–posttest study by Dompas et al. [35] referred to ‘an effect’. In this study, honey was consumed, combined with boiled water and betel leaves. This study did not examine whether the results were significant.

Regarding the intensity of pain, no significant differences were found in the RCTs of Gerosa et al., Nikpour et al., and Lavaf et al. [32,38,41]. However, 93% of the women in the study of Gerosa et al. were (very) satisfied with the use of honey. They found a significant decreases in the use of ibuprofen on day 4 in favor of the honey group despite the effects on pricking sensation being statistically non-significant. Lavaf did not find differences in the intake of pain relief drugs. However, Jaiswal et al. found significantly lower mean pain scores and less tenderness in the honey group after treatment when compared to before treatment [37]. In line with these findings, Pal et al. also demonstrated significantly reduced perineal pain, and less tenderness by honey [42].

**Table 3:** Summary of key outcomes per study. S = significant effect in favor of honey; NS = no significant difference; + = positive effect reported without significance mentioned (no control group).

Study	Wound Healing	Pain Intensity	Pain Relief Drugs Used	Pricking Sensation
Dompas, R. [35]	+			
Gerosa, D. [32]		NS	S	NS
Ghaderbasti, S. [36]	S			
Jaiswal, N. [37]	+	+		+
Lavaf, M. [38]	S	NS	NS	
Manjula, P. [39]	S			
Mulyaningsih, S. [40]	S			
Nikpour, M. [41]	NS	NS		
Pal, S. [42]	+	+		
Torkashvand, S. [43]	S			

## 3. Discussion

Many studies support the effectiveness of honey in wound management, for instance, in burns, diabetic foot ulcers, and chronic wounds [31,44,45]. Some studies indicate that honey can help reduce pain after tonsillectomy and in cold sores [46,47]. In this review, 10 studies were included, with a total of *n* = 723 participants who received an episiotomy or had a perineal tear. These studies have provided insight into the efficacy of honey on perineal trauma. Nine of the ten included studies found a positive effect of honey in at least one of the outcome measures. Wound healing was improved in eight out of nine studies, pain intensity was improved in two out of five studies, and pricking sensation and use of pain relief drugs improved in one out of two studies. Thus, it is found to accelerate recovery and improve the patient’s quality of life. In line with the conclusions of most of the included studies, the use of honey for the treatment of perineal wounds can be recommended.

Despite the consistent findings in wound healing and sometimes positive outcomes in other parameters, it is hard to draw an overarching conclusion, and the results should be interpreted with caution. There are many variables in the study setup, intervention, control groups, evaluated time points, and outcome parameters. Two studies investigated the effect of consuming honey [35,40], while the other studies investigated the effect of local application. Not only does the application of honey vary, but the composition of honey does as well. For example, pure honey [39], a mixture of honey with olive oil and sesame oil [43], 80% Manuka honey with 20% wax [32], 30–35% honey [38,41], honey in combination with boiled betel leaves [35], and pineapple juice with honey [40] were used in the studies considered. A higher percentage of honey does not necessarily lead to a more significant improvement in wound healing. Even with lower concentrations of honey, such as 30%, significant improvements in wound healing are observed. Honey is a natural product and, due to differences in composition and quality, the biological activity of honey varies. In addition, there might have been some interaction between honey and other supplements; however, these comprehensive medical preparations can have an additive effect. More research into the chemical composition and identification of the bioactive compounds of honey or other supplements and their molecular targets may further improve the therapeutic action on vaginal trauma in the future. In contrast to regular honey, (supplemented) MGH is, at least, standardized and carefully checked to ensure its quality, safety, and efficacy, and it is considered more consistent in its activity [26,48]. Therefore, it is recommended that MGH be used in future studies.

Moreover, the overall quality of available evidence is limited. Many studies lack transparency in their presentation of the results and methods. For instance, in the results section of the study by Jaiswal et al. [37], several tables are presented without captions or clear explanations, which complicates the interpretation of the results. Some studies are less reliable due to the lack of clearly formulated inclusion and exclusion criteria, for instance in the studies of Mulyaningsih et al. [40] and Dompas et al. [35] In addition, only three RCTs were double-blinded [36,38,41]. The other studies were (probably) not blinded, or single-blind, because no information was given about blinding. Also, unreliable study designs are included, such as the one-group pretest–posttest design [35], and the quasi-experimental design [40]. Quasi-experimental designs are susceptible to confounding variables as there is a lack of randomization, which can lead to bias. The findings of these study designs have a higher chance of being influenced by external factors.

Also, multiple studies lacked a proper control group. For example, when comparing the findings after treatment to the baseline level (before treatment) [35,37,42]. For wound healing, pain, and other outcomes, maturation effects are probable, because of expected improvement over time. In addition, the control group of the included studies varied from no intervention to placebo, standard hospital care, or another non-conventional treatment. Standard care can differ between hospitals. Because there is no consistent control group across all studies, no overarching conclusions can be drawn. The studies conducted by Nikpour et al., Lavaf et al., and Ghaderbasti et al. compared honey with a placebo [36,38,41]. However, it is very challenging to create a placebo for honey, due to its distinctive texture and scent. This raises questions about the integrity of the blinding process, potentially introducing bias. In addition, a placebo can also have some activity, e.g., by creating a moist wound environment. This might be a possible explanation as to why no significant differences were found in the study by Nikpour et al.

In most studies where honey was applied topically, it was used twice daily for a duration of 3 to 14 days, mostly for 10 days. Only in the study of Torkashvand et al. [43] was it used every 8 h, and in the study of Lavaf et al. [38] honey is applied once daily. It cannot be concluded that applying honey more frequently leads to a greater significant improvement. Only two studies reported the dosing (5 mL and one knuckle of cream). The use of clear application protocols and standardized honey formulations should help to ensure consistent results in future studies.

Some studies used a small sample size of 20 or 30 participants [35,37,42]. Small sample sizes can lead to less precise, less powerful, and more biased results. The ten studies were conducted in only four countries: Iran, Switzerland, Indonesia, and India. Four of these studies were performed in Iran, and three in India. This can negatively impact the reliability of the findings because of potential cultural and socioeconomic biases.

Many of the included studies did not distinguish between perineal tears versus episiotomies. Therefore, it is not possible to draw reliable conclusions about the differential effects of honey on the type of perineal trauma. It is important to draw this distinction, because there may be a difference in healing between perineal tears and episiotomy wounds.

The strength of this review is that it offers a comprehensive overview of the current literature concerning the use of honey for episiotomy wounds and perineal tears regarding wound healing and perineal pain. This patient group is considered homogeneous, as all individuals are postpartum women within a similar age range. This review includes a wide range of recent studies, with different study designs. Due to the systematic approach of our search, the extensivity of the search terms, and the search being performed in six different databases (and using additional sources), we expect that all relevant papers were found and included in this review.

To our knowledge, no previous literature review has specifically focused on perineal trauma and honey. However, Barbosa et al. conducted a systematic review recently (2024) investigating the effects of honey on wound healing and pain relief in obstetric wounds, including cesarean sections, episiotomies, and perineal tears, based exclusively on randomized controlled trials [49]. Surprisingly, they included only two RCTs with perineal wounds. Our review included six RCTs and other study designs. In addition, this review focused on perineal trauma, as cesarean sections and perineal injuries are not comparable, particularly due to differences in location, structure, and the microbiome. Barbosa et al. [49] concluded that honey has no significant improvement in wound healing, but that it is effective in reducing pain and decreasing the use of pain control medication.

This study is a good foundation for further research. Future studies are recommended to use MGH in (double)blinded randomized controlled trials. A power calculation is required to determine the sample size. Also, more research about adverse events, patient satisfaction, and overall experiences with honey treatment is warranted. Other outcome measures that should be examined include the effect of honey on scar formation, dyspareunia, infection and dehiscence, psychological well-being, and healthcare costs. MGH has been found effective in preventing infections previously in other studies, e.g., for the treatment of lacerations and C-sections [50,51,52]. It would be interesting to investigate the prophylactic activity of MGH for perineal wounds in the future.

## 4. Materials and Methods

### 4.1. Search Strategy

A literature search was performed on 17 July 2024 in databases PubMed, Embase, Cochrane, EBSCO host/CINAHL, Web of Science, and Scopus. Additionally, a search was performed on Google Scholar, and the reference lists of all the included studies were reviewed manually for studies that were eligible for inclusion (“snowballing”). The literature search included all studies investigating the effect of honey on episiotomy wounds or perineal tears. MeSH-terms and free-text terms that were used are, for example, (episiotomy) [MeSH] OR (episiotomy*) OR (perineotom*) OR (“perineal tear*”) OR (“perineal laceration*”) OR (“vaginal tear*”) AND (honey) [MeSH]. The full searches conducted in the different databases can be seen in ‘Appendix A’. Filters were not added regarding language or date of publication.

### 4.2. Study Selection

After obtaining all references, duplicates were removed. The first selection was based on the title and abstract. Next, the full texts were retrieved and assessed to see if they met the inclusion and exclusion criteria. The inclusion criteria were as follows: (1). studies with postpartum women who had a vaginal delivery and received an episiotomy, or had a perineal tear; (2). treatment with honey, compared to (no) control group or placebo; (3). the outcome measure being pain or wound healing. Exclusion criteria were as follows: (1). no episiotomy or perineal tear; (2). no intervention with honey; (3). there is no possibility of extracting data. The search included studies and articles published in any language. Whenever necessary, studies not written in English were translated with ‘Google Translate’. All articles were reviewed by one reviewer.

### 4.3. Data Extraction

The following data are extracted from the included studies: author, year of publication, research country, trial design, sample size, the inclusion-and-exclusion criteria, intervention and type of honey that was used, control group, dosage and frequency of treatment, outcome measures, results, and conclusion. Standardization of the study characteristics is difficult due to the diversity of research aims, study setup, and differences in reported outcome measures. However, we have unbiased and concisely presented as much relevant information as possible.

## 5. Conclusions

The findings of this review suggest that honey potentially has a great effect on wound healing and pain intensity of perineal trauma, as several studies show promising outcomes. However, the overall quality of available evidence is limited. Therefore, additional research, preferably in well-described large RCTs using MGH, is needed to determine the efficacy of honey in perineal trauma and to establish guidelines for clinical use. Performing cost-efficacy studies and investigating the prophylactic activity of MGH would be of additional interest.

## Figures and Tables

**Table 1:** REEDA scoring system. Total score of 0 = healed; 1–5 = moderately healed; 6–10 = mildly healed; 11–15 = not healed. S = significant improvement in at least one interval, NS = non-significant effect.

Score	Redness	Edema	Ecchymosis	Discharge	Approximation
**0**	None	None	None	None	Closed
**1**	<0.25 cm of incision bilaterally	Perineal <1 cm from incision	<0.25 cm bilaterally or 0.5 cm unilaterally of incision	Serous	Skin separation <3 mm
**2**	<0.5 cm of incision bilaterally	Perineal and/or 1–2 cm from incision	<0.25–1 cm bilaterally or 0.5–2 cm unilaterally of incision	Serosanguineous	Skin and subcutaneous fat separation
**3**	>0.5 cm of incision bilaterally	Perineal and/or vulvar >2 cm from incision	>1 cm bilaterally or 2 cm unilaterally of incision	Bloody purulent	Skin, subcutaneous fat, and fascial layer separation

**Table 2:** Overview of study characteristics and findings.

Selected Study, Year of Publication and Country	Trial Design and Sample Size	In- and Exclusion Criteria	Intervention	Control	Dosage/ Frequency of Treatment	Outcomes	Results/ Conclusion
Dompas et al. (2022), Indonesia [35]	One-group pretest–posttest design, *n* = 20	Not mentioned.	Group 1: 5 sheets of betel leaves boiled with 1000 mL water for 30 min to 600 mL, with pure honey.	No control group	Consuming twice a day (morning and evening) after bathing, for one week	**Wound healing *n* (%) Week 1:** Healed: 12/20 (60) Not healed: 8/20 (40)	There is an effect of green betel leaf water and pure honey on perineal wound healing in postpartum women.
Gerosa et al. (2022), Switzerland [32]	Randomized Controlled Trial (RCT), *n* = 68	**Inclusion:** >18 years, undergoing vaginal birth, speaking French, episiotomies or first- or second-degree perineal tears. **Exclusion:** third- or fourth-degree tears, postpartum hemorrhage >500 mL, allergy to honey or bee venom.	Group 1 (H): Medihoney (80% manuka honey, 20% wax) and standard maternity care.	Group 2 (C): only standard maternity care	Application on episiotomy wound after toileting practices, at least twice a day for 5 days postpartum.	**H vs. C** **Pain intensity, Mean VAS (SD)** Day (D)1: VAS 3.34 (2.35) vs. 3.38 (2.14), *p* = 0.65 D4: VAS 1.41 (1.49) vs. 2.28 (1.96), *p* = 0.09 **Qualitative pain: Questionnaire de Douleur Saint-Antoine. Mean intensity score (SD)** D1: 9.55 (7.03) vs. 8.63 (7.64), *p* = 0.42 D4: 3.93 (3.90) vs. 6.3 (7.94), *p* = 0.46 **Burning sensation during urination Mean VAS (SD)** D1: 2.64 (2.52) vs. 2.42 (2.18), *p* = 0.99 D4: 1.32 (1.93) vs. 1.73 (2.07), *p* = 0.31 **Woman who used pain relief drugs *n* (%)** D1: 23/29 (79.31) vs. 26/31 (83.87), *p* = 0.75 D4: 13/28 (46.43) vs. 17/31 (54.84), *p* = 0.61 **Mean ibuprofen use mg (SD)** D1: 707.14 (614.59) vs. 646.67 (537.38), *p* = 0.79 D4: 192.59 (339.60) vs. 466.67 (539.05), *p* = 0.049	There was no significant difference regarding pain intensity on day 1. On day 4, the VAS was 1.41 in the honey group versus 2.28 in the control group. However, this was also not significant (*p* = 0.09). A total of 93% of the women were (very) satisfied with the use of honey. There was a significant decrease in the use of ibuprofen on day 4 in favor of the honey group.
Ghaderbasti (2022), Iran [36]	Double-blind RCT *n* = 165	**Inclusion:** primiparous women with a singleton pregnancy, gestational age of 37–42 weeks, cephalic presentation, BMI of 19.8–26, spontaneous vaginal delivery, and mediolateral episiotomy. No history of previous injury or surgery and visible lesions of the perineum, no continuous attachment, no rupture of the water sac for >18 h, no abnormal bleeding after delivery, no manual removal of the placenta, no perineal hematoma. **Exclusion:** unwillingness to continue participating in the study, incorrect use of creams, allergy to honey or olive oil, sexual intercourse in the first 10 days after delivery, history of diabetes, hypertension, cardiovascular or respiratory diseases, or any chronic illness affecting wound healing.	Group 1 (H): natural honey cream, with 0.2 paraben added as a preservative. Group 2 (O): pure olive oil Lanolin alcohol was used as the base of the creams.	Group 3 (C): placebo cream with the same weight, color, shape, and smell as the intervention groups.	Application on episiotomy wound every 12 h, up to 10 days postpartum.	**H vs. O vs. C** **Wound healing. Mean REEDA (SD)** Baseline: 4.76 (0.94) vs. 4.78 (1.03) vs. 4.82 (0.81), *p* = 0.955 D5: 2.64 (0.88) vs. 2.98 (1.01) vs. 5.27 (1.19), *p* = 0.0001 D10: 0.64 (0.63) vs. 1.04 (1.25) vs. 2.90 (0.74), *p* = 0.0001	The honey group had a significantly greater reduction in mean REEDA score, compared to the olive oil group. However, olive oil also showed positive effects.
Jaiswal et al. (2019), India [37]	Single-blind comparative clinical study. *n* = 30	**Inclusion:** both primiparous and multiparous women aged 18–40 years, undergoing normal vaginal delivery with an episiotomy wound. **Exclusion:** abnormal labor, third- or fourth-degree perineal tear, any complications like cervical tear or postpartum hemorrhage, systemic illness like diabetes mellitus, hypertension, etc.	Group 1 (H): Sharapunkha Moola twak kalka with honey (cream). Group 2 (J): Jatyadi taila (herb oil).	No control group	Application on episiotomy wound for three days. Dosage and frequency of application were not mentioned.	**Perineal pain (D3):** H: Mean negative rank (MR) = 7.5. Sum of negative ranks (SR) = 105, *p* = 0.000 J: MR = 7.0 SR = 91, *p* = 0.000 **Pricking sensation (D3):** H: MR = 3 SR = 15, *p* = 0.034 J: MR = 1.5. SR = 3, *p* = 0.157 **Tenderness (D3):** H: MR = 5 SR = 45, *p* = 0.006 J: MR = 7.65. SR = 99.5, *p* = 0.002 Wound healing (REEDA scale) **Redness (D3):** H: MR = 3.5. SR = 21, *p* = 0.024 J: MR = 3. SR = 15, *p* = 0.038 **Edema (D3):** H: MR = 3.5. SR = 21. *p* = 0.014 J: MR = 2. SR = 6. *p* = 0.102	In group 1, before- and after treatment, statistically significant results were obtained regarding pain (*p* = 0.000), pricking sensation (*p* = 0.034), tenderness (*p* = 0.006), redness (*p* = 0.034) and edema (*p* = 0.014). In group 2, before and after treatment, statistically significant results were obtained regarding pain (*p* = 0.000), tenderness (*p* = 0.002) and redness (*p* = 0.038), but not for pricking sensation (0.157) and edema (*p* = 0.102).
Lavaf et al. (2017), Iran [38]	Double blind-three group RCT *n* = 120	**Inclusion:** among others, Iranian nationality, nulliparity, age 18–35 years, pregnancy with singleton live fetus and cephalic presentation, newborn weight of 2500–4000 gr, BMI 19.8–30, not taking medications and psychotropic drugs, not developing wound healing disruptive disorders, and no previous damages from operations in perineal region, no rupture of amniotic membrane for >18 h. **Exclusion:** using healing medications, lack of regular cream-usage according to prescription, allergy to cream, refusal of participation, having sexual intercourse in the first 5 days postpartum, refusing to attend the hospital on the 7th and 15th day.	Group 1 (H): natural honey from the Qamsar region. Concentration of 30%. Group 2 (Ph): sodium Phenytoin cream 1%.	Group 3 (C): placebo in a metal 30 g tube	Application of one knuckle of cream on episiotomy wound, once, at night, for 10 days.	**H vs. Ph vs. CE** **pisiotomy wound healing. (Total REEDA score), *n* (%), D7:** (score 0): 16 (48.4) vs. 14 (38.8) vs. 10 (26.3) (score 1–2): 15 (45.5) vs. 19 (52.7) vs. 20 (52.6) (score 3–5): 2 (6.1) vs. 3 (8.5) vs. 8 (21.1) *p* = 0.011 H vs. C with Mann–Whitney U test and Bonferroni correction: *p* = 0.005 **Wound discharge. *n* (%), D7:** No discharge: 28 (84.8) vs. 30 (83.3) vs. 21 (55.2) Serous: 5 (15.2) vs. 6 (16.7) vs. 15 (39.4) Bloody plasma: 0 vs. 0 vs. 1 (2.7) Septic–bloody: 0 vs. 0 vs. 1 (2.7) *p* = 0.004 Mann–Whitney U test H vs. C: *p* = 0.008 **Pain intensity [numeric scale], *n* (%), D7:** No pain [0]: 10 (30.5) vs. 4 (11.1) vs. 7 (18.4) Mild pain [1–3]: 11 (33.3) vs. 20 (55.5) vs. 14 (36.8) Middle pain [4–7]: 6 (18.1) vs. 10 (27.7) vs. 13 (34.3) Severe pain [8–10]: 6 (18.1) vs. 2 (5.7) vs. 4 (10.5) *p* = 0.8 **D14:** No pain [0]: 25 (75.7) vs. 28 (81.3) vs. 21 (55.2) Mild pain [1–3]: 4 (12.1) vs. 5 (10.8) vs. 10 (26.4) Middle pain [4–7]: 4 (12.1) vs. 3 (8.3) vs. 7 (18.4) Severe pain [8–10]:0 vs. 0 vs. 0 *p* = 0.19	There was a significant difference in total REEDA score between honey and the control group on the 7th day. There was also a significant difference in episiotomy wound discharge between honey and the control group. There was no significant difference between the three groups regarding pain intensity on the 7th or 14th day. There was no significant difference between the three groups regarding times of consumption of pain relief drugs.
Manjula et al. (2012), India [39]	Unblinded RCT *n* = 61	**Inclusion:** postnatal women aged >18 years with right or left mediolateral episiotomy. **Exclusion:** not mentioned.	Group 1 (H): pure, natural honey without added sugar.	Group 2 (B): betadine	Application 5 mL of honey soaked in gauze to the episiotomy wound every 12 h until 5 days postpartum.	**H vs. B** **Wound healing. Mean episiotomy wound assessment score 0–18 (SD).** Baseline: 8.07 (2.27) vs. 8.23 (1.31), *p* = 0.729 D2: 4.53 (2.15) vs. 6.63 (1.94), *p* = 0.000 D3: 2.50 (1.82) vs. 3.93 (1.76), *p* = 0.003 D4: 0.90 (0.92) vs. 2.37 (1.59), *p* = 0.000 D5: 0.27 (0.64) vs. 1.40 (1.33), *p* = 0.000	There is a statistically significant difference between the healing pace of the honey group (faster) and the betadine group.
Mulyaningsih et al. (2021), Indonesia [40]	Quasi-experimental design, posttest-only control group design. *n* = 36	Not mentioned.	Group 1 (H): pineapple juice and honey.	Group 2 (C): no treatment	Consuming 150 mL pineapple juice and honey twice a day for 7 days.	**H vs. C** **Frequency healed wounds *n* (%)** Healed: 15/18 (83.3) vs. 3/18 (16.7) **Wound healing mean (SD)** (outcome measurement not specified) 24.50 (507) vs. 12.50 (507) Mann–Whitney U test *p* = 0.000	Pineapple juice and honey had a significant effect on the acceleration of perineal wound healing.
Nikpour et al. (2019), Iran [41]	Double-blind three-group RCT. *n* = 120	**Inclusion:** age of 17–35 years, gestational age of 37–42 weeks, no smoking or drug abuse, no health conditions or medications that could negatively affect wound healing, no perineal hematoma, no abnormal vaginal bleeding, no third- or fourth-degree perineal tear, and no infantile hospitalization for >7 days. **Exclusion:** using the allocated treatment irregularly, failing to refer to the study setting for follow-up visits and assessments.	Group 1 (H): 35% honey. Group 2 (Cu): Curcumin 2%.	Group 3 (C): placebo containing glycerin, Carbopol, Eucerin triethanolamine, propylparaben, distilled water, and food dye.	Application on episiotomy wound twice daily for 10 postpartum days.	**H vs. Cu vs. C** **Pain intensity. Mean (SD)** 2 h: 4.20 (2.42) vs. 3.80 (2.04) vs. 4.14 (2.27), *p* = 0.762 D5: 2.83 (1.72) vs. 3.90 (2.40) vs. 3.69 (2.17), *p* = 0.121 D10: 1.36 (1.24) vs. 2.06 (2.06) vs. 1.96 (1.42), *p* = 0.202 Group: *p* = 0.253 **Wound healing. Mean REEDA (SD)** 2 h: 2.30 (1.60) vs. 2.47 (1.40) vs. 2.79 (1.84) *p* = 0.498 D5: 2.00 (1.50) vs. 2.47 (1.25) vs. 2.21 (1.17) *p* = 0.395 D10: 1.73 (1.41) vs. 1.63 (1.27) vs. 1.83 (1.10) *p* = 0.842 Group: *p* = 0.547	There was no significant difference among the three groups regarding wound healing or pain intensity.
Pal et al. (2017), India [42]	Comparative clinical study. *n* = 30	**Inclusion:** women aged 20–35 years, who underwent normal vaginal delivery with episiotomy or first- or second-degree perineal tear, patients who had regular antenatal checkups and previous routine investigations performed regularly. **Exclusion:** severe anemia, third degree perineal tear, any skin disease, allergic reaction, positive VDRL (Venereal Disease Research Laboratory test), HIV, HbsAg, systemic illness like diabetes mellitus, tuberculosis, thyroid dysfunction, hypertension, etc.	Group 1 (H): Nimbadi ointment, containing Nimba, Daruharidra, Yastimadhu, and Tila; the base is honey and ghee. Group 2 (Y): Yastyadi ointment, containing Yastimadhu, and Tila; the base is ghee.	No control group.	Application on the episiotomy wound twice a day for 14 days.	**Before treatment vs. after treatment** **Pain. Mean (SD)** H: 1.53 vs. 0.666 (0.833), *p* = 0.002 Y: 1.46 vs. 1.00 (0.51), *p* = 0.15 **Pricking sensation. Mean (SD)** H: 0.53 vs. 0.13 (0.50), *p* = 0.031Y: 0.73 vs. 0.26 (0.51), *p* = 0.15 **Tenderness Mean (SD)** H: 1.13 vs. 0.533 (0.632), *p* = 0.07 Y: 1.4 vs.1.0 (0.507), *p* = 0.0313 **Redness. Mean (SD)** H: 0.896 vs. 0.466 (0.56), *p* = 0.002 Y: 1.26 vs. 0.733 (0.51), *p* = 0.007 **Edema. Mean (SD)** H: 0.6 vs. 0.06 (0.83), *p* = 0.031 Y: 0.6 vs. 0.2 (0.63), *p* = 0.062 **Ecchymosis. Mean (SD)** H: 0.93 vs. 0.33 (0.50), *p* = 0.003 Y: 1.26 vs. 0.80 (0.51), *p* = 0.01 **Discharge. Mean (SD)** H: 0.46 vs. 0.06 (0.56), *p* = 0.5 Y: 0.33 vs. 0.066 (0.70), *p* = 0.05 **Approximation Mean (SD)** H: 0.20 vs. 0.06 (0.35), *p* = 0.50 Y: 0.26 vs. 0.13 (0.35), *p* = 0.50 The average improvement was 66.6% (group 1), compared to 49,8% (group 2) (all parameters)	Group 1 and 2 are not compared. In group 1, before- and after treatment, highly significant results were obtained regarding the relief of pain, tenderness, redness, and ecchymosis, and significant results were obtained regarding pricking sensation and edema. Discharge and approximation were insignificant. The average improvement was better in the Nimbadi-group than the Yastyadi-group.
Torkashvand et al. (2021), Iran [43]	RCT *n* = 73	**Inclusion:** among others, Iranian nationality, primiparous, age of 18–35 years, live term singleton pregnancy with vertex presentation, mediolateral episiotomy with no rupture, no chronic systemic diseases, no history of previous injury, surgery and visible lesions in the perineum. **Exclusion:** among others, reluctance to further participate in the study, use of other supplements for wound healing, incorrect usage of the ointment for >2 nights, sensitivity to Olea ointment.	Group 1 (H): olea ointment: herbal mixture of honey. (33.4%), olive oil (33.3%), and sesame oil (33.3%).	Group 2 (C): only routine hospital care as washing with normal saline twice per day until the 10th postpartum day.	Application on episiotomy wound at 4 h post-episiotomy and afterwards every 8 h until the 10th postpartum day.	**H vs. C** **Wound healing. Mean REEDA (SD)** Baseline: 2.72 (0.46) vs. 2.71 (0.46), *p* = 0.91 2 h: 2.31 (0.47) vs. 2.65 (0.49), *p* = 0.004 24 h: 1.67 (0.62) vs. 2.29 (0.46), *p* < 0.001 D5: 0.05 (0.22) vs. 0.35 (0.49), *p* = 0.001 D10: 0.00 vs. 0.29 (0.46), *p* < 0.001	There was a significant difference in episiotomy wound healing at four intervals between the Olea ointment and control group.

## Data Availability

No new data were created or analyzed in this study. Data sharing is not applicable to this article.

## Appendix A. Full Literature Searches

The search was performed on 17 July 2024 on five different databases.

*Pubmed:*

(((((((((((((episiotomy[MeSH Terms]) OR (episiotom* [tiab])) OR (perineotom* [tiab])) OR (perineal tear* [tiab])) OR (perineal laceration* [tiab])) OR (perineal injur* [tiab])) OR (perineal trauma* [tiab])) OR (obstetric tear* [tiab])) OR (obstetric laceration* [tiab])) OR (vaginal tear* [tiab])) OR (vaginal laceration* [tiab])) OR (childbirth tear* [tiab])) OR (childbirth laceration* [tiab])) AND (((((((((((((((medical grade honey [tiab]) OR (therapeutic honey [tiab])) OR (medicinal honey [tiab])) OR (honey therap* [tiab])) OR (honey treatment [tiab])) OR (antibacterial honey [tiab])) OR (antimicrobial honey [tiab])) OR (medihoney [tiab])) OR (mesitran [tiab])) OR (manuka honey [tiab])) OR (leptospermum honey [tiab])) OR (tualang honey [tiab])) OR (honey [tiab])) OR (surgihoney [tiab])) OR (activon [tiab]))

Total hits: 5.

*Cochrane Library:*

1. ((episiotom*) OR (perineotom*) OR (perineal NEXT tear*) OR (perineal NEXT laceration*) OR (perineal NEXT injur*) OR (perineal NEXT trauma*) OR (obstetric NEXT laceration*) OR (vaginal NEXT tear*) OR (vaginal NEXT laceration*) OR (childbirth NEXT tear*) OR (childbirth NEXT laceration*)) (Word variations have been searched)
2. ((“medical grade honey”) OR (“medicinal honey”) OR (honey NEXT therap*) OR (“honey treatment”) OR (“antibacterial honey”) OR (“antimicrobial honey”) OR (“medihoney”) OR (“mesitran”) OR (“manuka honey”) OR (“leptospermum honey”) OR (“tualang honey”) OR (“honey”) OR (“surgihoney”) OR (“activon”)) (Word variations have been searched)
3. #1 AND #2

Total hits: 12.

*Scopus:*

((TITLE-ABS-KEY (“medical grade honey”)) OR (TITLE-ABS-KEY (“therapeutic honey”)) OR (TITLE-ABS-KEY (“medicinal honey”)) OR (TITLE-ABS-KEY (“honey therap*”)) OR (TITLE-ABS-KEY (“honey treatment”)) OR (TITLE-ABS-KEY (“antibacterial honey”)) OR (TITLE-ABS-KEY (“antimicrobial honey”)) OR (TITLE-ABS-KEY (medihoney)) OR (TITLE-ABS-KEY (mesitran)) OR (TITLE-ABS-KEY (“manuka honey”)) OR (TITLE-ABS-KEY (“tualang honey”)) OR (TITLE-ABS-KEY (“honey”)) OR (TITLE-ABS-KEY (surgihoney)) OR (TITLE-ABS-KEY (“leptospermum honey”)) OR (TITLE-ABS-KEY (activon))) AND ((TITLE-ABS-KEY (“childbirth laceration*”)) OR (TITLE-ABS-KEY (“childbirth tear*”)) OR (TITLE-ABS-KEY (“vaginal laceration*”)) OR (TITLE-ABS-KEY (“vaginal tear*”)) OR (TITLE-ABS-KEY (“obstetric laceration*”)) OR (TITLE-ABS-KEY (“obstetric tear*”)) OR (TITLE-ABS-KEY (“perineal trauma*”)) OR (TITLE-ABS-KEY (“perineal injur*”)) OR (TITLE-ABS-KEY (“perineal laceration*”)) OR (TITLE-ABS-KEY (“perineal tear*”)) OR (TITLE-ABS-KEY (perineotom*)) OR (TITLE-ABS-KEY (episiotom*)))

Total hits: 11.

*Web of Science:*

4. (TS = (episiotom*)) OR (TS = (perineotom*)) OR (TS = (“perineal tear*”)) OR (TS = (“perineal laceration*”)) OR (TS = (“perineal injur*”)) OR (TS = (“perineal trauma*”)) OR (TS = (“obstetric tear*”)) OR (TS = (“obstetric laceration*”)) OR (TS = (“vaginal tear*”)) OR (TS = (“vaginal laceration*”)) OR (TS = (“childbirth laceration*”))
5. (TS = (“therapeutic honey”)) OR (TS = (“medical grade honey”)) OR (TS = (“medicinal honey”)) OR (TS = (“honey therap*”)) OR (TS = (“honey treatment”)) OR (TS = (“antibacterial honey”)) OR (TS = (“antimicrobial honey”)) OR (TS = (medihoney)) OR (TS = (mesitran)) OR (TS = (“manuka honey”)) OR (TS = (“leptospermum honey”)) OR (TS = (“honey”)) OR (TS = (“tualang honey”)) OR (TS = (surgihoney)) OR (TS = (activon))
6. #1 AND #2

Total hits: 10.

*EBSCO host/CINAHL:*

7. (TI episiotom* OR AB episiotom* OR MH episiotom*) OR (TI perineotom* OR AB perineotom* OR MH perineotom*) OR (TI “perineal tear*” OR AB “perineal tear*” OR MH “perineal tear*”) OR (TI “perineal trauma*” OR AB “perineal trauma*” OR MH “perineal trauma*”) OR (TI “perineal laceration*” OR AB “perineal laceration*” OR MH “perineal laceration*”) OR (TI “perineal injur*” OR AB “perineal injur*” OR MH “perineal injur*”) OR (TI “obstetric tear*” OR AB “obstetric tear*” OR MH “obstetric tear*”) OR (TI “obstetric laceration*” OR AB “obstetric laceration*” OR MH “obstetric laceration*”) OR (TI “vaginal tear*” OR AB “vaginal tear*” OR MH “vaginal tear*”) OR (TI “vaginal laceration*” OR AB “vaginal laceration*” OR MH “vaginal laceration*”) OR (TI “childbirth tear*” OR AB “childbirth tear*” OR MH “childbirth tear*”) OR (TI “childbirth laceration*” OR AB “childbirth laceration*” OR MH “childbirth laceration*”)
8. (TI “medical grade honey” OR AB “medical grade honey” OR MH “medical grade honey”) OR (TI “therapeutic honey” OR AB “therapeutic honey” OR MH “therapeutic honey”) OR (TI “medicinal honey” OR AB “medicinal honey” OR MH “medicinal honey”) OR (TI “honey therap*” OR AB “honey therap*” OR MH “honey therap*”) OR (TI “honey treatment” OR AB “honey treatment” OR MH “honey treatment”) OR (TI “antibacterial honey” OR AB “antibacterial honey” OR MH “antibacterial honey”) OR (TI “antimicrobial honey” OR AB “antimicrobial honey” OR MH “antimicrobial honey”) OR (TI medihoney OR AB medihoney OR MH medihoney) OR (TI mesitran OR AB mesitran OR MH mesitran) OR (TI “manuka honey” OR AB “manuka honey” OR MH “manuka honey”) OR (TI “leptospermum honey” OR AB “leptospermum honey” OR MH “leptospermum honey”) OR (TI “tualang honey” OR AB “tualang honey” OR MH “tualang honey”) OR (TI honey OR AB honey OR MH honey) OR (TI surgihoney OR AB surgihoney OR MH surgihoney) OR (TI activon OR AB activon OR MH activon)
9. S1 AND S2

Total hits: 5.

*Embase:*

10. episiotom*.ti,ab,kf.
11. perineotom*.ti,ab,kf.
12. “perineal tear*”.ti,ab,kf.
13. “perineal laceration*”.ti,ab,kf.
14. “perineal trauma*”.ti,ab,kf.
15. “obstetric tear*”.ti,ab,kf.
16. “obstetric laceration*”.ti,ab,kf.
17. “vaginal tear*”.ti,ab,kf.
18. “childbirth tear*”.ti,ab,kf.
19. “childbirth laceration*”.ti,ab,kf.
20. 1 or 2 or 3 or 4 or 5 or 6 or 7 or 8 or 9 or 10
21. “medical grade honey”.ti,ab,kf.
22. “therapeutic honey”.ti,ab,kf.
23. “medicinal honey”.ti,ab,kf.
24. “honey therap*”.ti,ab,kf.
25. “honey treatment”.ti,ab,kf.
26. “antibacterial honey”.ti,ab,kf.
27. “antimicrobial honey”.ti,ab,kf.
28. medihoney.ti,ab,kf.
29. mesitran.ti,ab,kf.
30. “manuka honey”.ti,ab,kf.
31. “leptospermum honey”.ti,ab,kf.
32. “tualang honey”.ti,ab,kf.
33. honey.ti,ab,kf.
34. surgihoney.ti,ab,kf.
35. activon.ti,ab,kf.
36. 12 or 13 or 14 or 15 or 16 or 17 or 18 or 19 or 20 or 21 or 22 or 23 or 24 or 25 or 26
37. 11 and 27

Total hits: 6.

*Google Scholar:*

“Episiotomy wounds” AND “honey”.

Total hits: 938.

Until page 41.

## References

[1] Frohlich J., Kettle C. (2015). Perineal care. BMJ Clin. Evid..

[2] Opondo C., Harrison S., Sanders J., Quigley M.A., Alderdice F. (2023). The relationship between perineal trauma and postpartum psychological outcomes: A secondary analysis of a population-based survey. BMC Pregnancy Childbirth.

[3] Smith L.A., Price N., Simonite V., Burns E.E. (2013). Incidence of and risk factors for perineal trauma: A prospective observational study. BMC Pregnancy Childbirth.

[4] Seijmonsbergen-Schermers A., Thompson S., Feijen-de Jong E., Smit M., Prins M., van den Akker T., de Jonge A. (2021). Understanding the perspectives and values of midwives, obstetricians and obstetric registrars regarding episiotomy: Qualitative interview study. BMJ Open.

[5] Perined (2021). Perinatale Zorg in Nederland Anno 2020: Duiding Door Landelijke Perinatale Audit en Registratie.

[6] (2018). ACOG Practice Bulletin No. 198: Prevention and Management of Obstetric Lacerations at Vaginal Delivery. Obstet. Gynecol..

[7] Barjon K., Vadakekut E.S., Mahdy H. (2024). Episiotomy. StatPearls.

[8] Carroli G., Mignini L. (2009). Episiotomy for vaginal birth. Cochrane Database Syst. Rev..

[9] Goh R., Goh D., Ellepola H. (2018). Perineal tears—A review. Aust. J. Gen. Pract..

[10] Okeahialam N.A., Sultan A.H., Thakar R. (2024). The prevention of perineal trauma during vaginal birth. Am. J. Obstet. Gynecol..

[11] Richtlijnendatabase Routinematig Episiotomie Bij Kunstverlossing. https://richtlijnendatabase.nl/richtlijn/totaalruptuur/preventie_van_een_ruptuur/routinematige_episiotomie_bij_kunstverlossing_2024.html.

[12] World Health Organization (2018). WHO Recommendations: Intrapartum Care for a Positive Childbirth Experience.

[13] Ramar C.N., Vadakekut E.S., Grimes W.R. (2024). Perineal Lacerations. StatPearls.

[14] Andrews V., Thakar R., Sultan A.H., Jones P.W. (2008). Evaluation of postpartum perineal pain and dyspareunia--a prospective study. Eur. J. Obstet. Gynecol. Reprod. Biol..

[15] Macarthur A.J., Macarthur C. (2004). Incidence, severity, and determinants of perineal pain after vaginal delivery: A prospective cohort study. Am. J. Obstet. Gynecol..

[16] Klein M.C., Gauthier R.J., Robbins J.M., Kaczorowski J., Jorgensen S.H., Franco E.D., Johnson B., Waghorn K., Gelfand M.M., Guralnick M.S. (1994). Relationship of episiotomy to perineal trauma and morbidity, sexual dysfunction, and pelvic floor relaxation. Am. J. Obstet. Gynecol..

[17] Vieira F., Guimaraes J.V., Souza M.C.S., Sousa P.M.L., Santos R.F., Cavalcante A. (2018). Scientific evidence on perineal trauma during labor: Integrative review. Eur. J. Obstet. Gynecol. Reprod. Biol..

[18] Kamel A., Khaled M. (2014). Episiotomy and obstetric perineal wound dehiscence: Beyond soreness. J. Obstet. Gynaecol..

[19] Jones K., Webb S., Manresa M., Hodgetts-Morton V., Morris R.K. (2019). The incidence of wound infection and dehiscence following childbirth-related perineal trauma: A systematic review of the evidence. Eur. J. Obstet. Gynecol. Reprod. Biol..

[20] Fenner D.E., Genberg B., Brahma P., Marek L., DeLancey J.O. (2003). Fecal and urinary incontinence after vaginal delivery with anal sphincter disruption in an obstetrics unit in the United States. Am. J. Obstet. Gynecol..

[21] LaCross A., Groff M., Smaldone A. (2015). Obstetric anal sphincter injury and anal incontinence following vaginal birth: A systematic review and meta-analysis. J. Midwifery Womens Health.

[22] Andreucci C.B., Bussadori J.C., Pacagnella R.C., Chou D., Filippi V., Say L., Cecatti J.G., Brazilian C.S.G., Group W.H.O.M.M.W. (2015). Sexual life and dysfunction after maternal morbidity: A systematic review. BMC Pregnancy Childbirth.

[23] Saikaly S.K., Khachemoune A. (2017). Honey and Wound Healing: An Update. Am. J. Clin. Dermatol..

[24] Scepankova H., Combarros-Fuertes P., Fresno J.M., Tornadijo M.E., Dias M.S., Pinto C.A., Saraiva J.A., Estevinho L.M. (2021). Role of Honey in Advanced Wound Care. Molecules.

[25] Minden-Birkenmaier B.A., Bowlin G.L. (2018). Honey-Based Templates in Wound Healing and Tissue Engineering. Bioengineering.

[26] Hermanns R., Mateescu C., Thrasyvoulou A., Tananaki C., Wagener F.A.D.T.G., Cremers N.A.J. (2020). Defining the standards for medical grade honey. J. Apic. Res..

[27] Oryan A., Alemzadeh E., Moshiri A. (2016). Biological properties and therapeutic activities of honey in wound healing: A narrative review and meta-analysis. J. Tissue Viability.

[28] Pleeging C.C.F., Coenye T., Mossialos D., de Rooster H., Chrysostomou D., Wagener F., Cremers N.A.J. (2020). Synergistic Antimicrobial Activity of Supplemented Medical-Grade Honey against Pseudomonas aeruginosa Biofilm Formation and Eradication. Antibiotics.

[29] Pleeging C.C.F., Wagener F., de Rooster H., Cremers N.A.J. (2022). Revolutionizing non-conventional wound healing using honey by simultaneously targeting multiple molecular mechanisms. Drug Resist. Updates.

[30] Aguiar R., Duarte F.C., Mendes A., Bartolome B., Barbosa M.P. (2017). Anaphylaxis caused by honey: A case report. Asia Pac. Allergy.

[31] Holubova A., Chlupacova L., Krocova J., Cetlova L., Peters L.J.F., Cremers N.A.J., Pokorna A. (2023). The Use of Medical Grade Honey on Infected Chronic Diabetic Foot Ulcers-A Prospective Case-Control Study. Antibiotics.

[32] Gerosa D., Santagata M., Martinez de Tejada B., Guittier M.-J. (2022). Application of Honey to Reduce Perineal Laceration Pain during the Postpartum Period: A Randomized Controlled Trial. Healthcare.

[33] Alvarenga M.B., Francisco A.A., de Oliveira S.M., da Silva F.M., Shimoda G.T., Damiani L.P. (2015). Episiotomy healing assessment: Redness, Oedema, Ecchymosis, Discharge, Approximation (REEDA) scale reliability. Rev. Lat. Am. Enferm..

[34] Armata N.N. REEDA: Episiotomy Healing Assessment Acronym. https://www.osmosis.org/answers/reeds-episiotomy-healing-assessment-acronym.

[35] Dompas R., Montolalu A., Koloay T.G. (2022). Green Betel Leaf Water with Pure Honey Against Healing Perineum’s Wounds Mother Postpartum. J. Midwifery.

[36] Ghaderbasti S., Kabuli K.S., Farhadifar F., Shahavi R. (2022). Comparative study of the effect of honey cream and olive oil cream on the healing rate of episiotomy. Iran. J. Women Midwifery Infertil.

[37] Jaiswal N., Ramadevi G. (2019). A Comparative Clinical Study Of Sharapunkha Moola Twak Kalka With Jatyadi Taila Application On Episiotomy Wound. World J. Pharm. Res..

[38] Lavaf M., Simbar M., Mojab F., Alavi Majd H., Samimi M. (2017). Comparison of honey and phenytoin (PHT) cream effects on intensity of pain and episiotomy wound healing in nulliparous women. J. Complement. Integr. Med..

[39] Manjula P., Rao A.C., Ranjani P. (2012). Effectiveness of Honey Versus Betadine on Episiotomy Wound Healing. I-Manag. J. Nurs..

[40] Mulyaningsih S., Dunggio R., Susanti K.A. (2020). The Effect of Pineapple Juice and Honey on the Acceleration of Perineal Wound Healing in Post-Partum Mothers in the Work Area of Dr. M.M. Dunda Limboto Hospital. J. Community Health Provis..

[41] Nikpour M., Delavar M.A., Khafri S., Ghanbarpour A., Moghadamnia A.A., Esmaeilzadeh S., Behmanesh F. (2019). The Use of Honey and Curcumin for Episiotomy Pain Relief and Wound Healing: A Three-Group Double-Blind Randomized Clinical Trial. Nurs. Midwifery Stud..

[42] Pal S., Pushpalatha B., Bharathi K. (2017). A comparative clinical study of nimbadi and yastyadi ointment on episiotomy wound W.S.R. to wound healing. Ayushdhara.

[43] Torkashvand S., Jafarzadeh-Kenarsari F., Donyaei-Mobarrez Y., Gholami Chaboki B. (2021). Effectiveness of Olea Herbal Ointment on Episiotomy Wound Healing Among Primiparous Women: A Randomized Clinical Trial. Jundishapur J. Nat. Pharm. Prod..

[44] Al-Waili N., Salom K., Al-Ghamdi A.A. (2011). Honey for wound healing, ulcers, and burns; data supporting its use in clinical practice. Sci. World J..

[45] Papanikolaou G.E., Gousios G., Cremers N.A.J. (2023). Use of Medical-Grade Honey to Treat Clinically Infected Heel Pressure Ulcers in High-Risk Patients: A Prospective Case Series. Antibiotics.

[46] Hwang S.H., Song J.N., Jeong Y.M., Lee Y.J., Kang J.M. (2016). The efficacy of honey for ameliorating pain after tonsillectomy: A meta-analysis. Eur. Arch. Oto-Rhino-Laryngol..

[47] Naik P.P., Mossialos D., Wijk B.V., Novakova P., Wagener F., Cremers N.A.J. (2021). Medical-Grade Honey Outperforms Conventional Treatments for Healing Cold Sores-A Clinical Study. Pharmaceuticals.

[48] Lardenoije C., van Riel S., Peters L.J.F., Wassen M., Cremers N.A.J. (2024). Medical-Grade Honey as a Potential New Therapy for Bacterial Vaginosis. Antibiotics.

[49] Ferraz Barbosa B., de Moraes F.C.A., Araujo Alves da Silva B., Bordignon Barbosa C., Pereira da Silva I., da Silva E.R., Barros J.C.M., Rebouças L.W.C., dos Santos N.P.C., Fernandes M.R. (2024). The Use of Honey for Cicatrization and Pain Control of Obstetric Wounds: A Systematic Review and Meta-Analysis of Randomized Controlled Trials. Nutrients.

[50] Mandel H.H., Sutton G.A., Abu E., Kelmer G. (2020). Intralesional application of medical grade honey improves healing of surgically treated lacerations in horses. Equine Vet. J..

[51] Bocoum A., Riel S., Traore S.O., Ngo Oum Ii E.F., Traore Y., Thera A.T., Fane S., Dembele B.T., Cremers N.A.J. (2023). Medical-Grade Honey Enhances the Healing of Caesarean Section Wounds and Is Similarly Effective to Antibiotics Combined with Povidone-Iodine in the Prevention of Infections-A Prospective Cohort Study. Antibiotics.

[52] Dryden M., Goddard C., Madadi A., Heard M., Saeed K., Cooke J. (2014). Using antimicrobial surgihoneyto prevent caesarean wound infection. Br. J. Midwifery.

